# Fermented cassava peel with *Pleurotus ostreatus* as a functional feed: Effects on growth performance, gut health, microbiota, and meat quality in broiler chickens

**DOI:** 10.14202/vetworld.2026.2434-2449

**Published:** 2026-06-13

**Authors:** Nuraini Nuraini, Jamila Mustabi, Denny Rusmana, Mirzah Mirzah, Ridho Kurniawan Rusli, M Habilburahman Habilburahman, Fauziah Zahratul Alfath, Muthia Rahmi Alfianti

**Affiliations:** 1 Department of Nutrition and Feed Technology, Faculty of Animal Science, Universitas Andalas, Padang, Indonesia; 2 Department of Nutrition and Feed Technology, Faculty of Animal Science, Universitas Hasanuddin, Makassar, Indonesia; 3 Department of Animal Nutrition and Feed Technology, Faculty of Animal Science, Padjajaran University, Sumedang, West Java, Indonesia; 4 Student of the Department of Nutrition and Feed Technology, Faculty of Animal Science, Universitas Andalas, Indonesia

**Keywords:** antioxidants, broiler chickens, cassava peel fermentation, gut microbiota, *Pleurotus ostreatus*, poultry nutrition, sustainable feed, β-glucan

## Abstract

**Background and Aim:**

The utilization of agro-industrial by-products as alternative feed ingredients has gained increasing attention in poultry production. Cassava peel is abundant but limited by high fiber and anti-nutritional factors. Fermentation using *Pleurotus ostreatus* can improve its nutritional quality and generate bioactive compounds. This study aimed to evaluate the effects of fermented cassava peel (FCP) on growth performance, carcass traits, immune response, intestinal morphology, microbial population, blood biochemical parameters, and fatty acid profile in broiler chickens.

**Materials and Methods:**

A total of broilers were randomly assigned to five dietary treatments: FCP0, FCP5, FCP10, FCP15, and FCP20, representing 0%, 5%, 10%, 15%, and 20% inclusion levels of FCP in the diet. The experiment was conducted over 35 days, including starter and finisher phases. Parameters measured included average daily weight gain (ADWG), average daily feed intake (ADFI), feed conversion ratio (FCR), live body weight (LBW), carcass characteristics, immune organ weights, intestinal villus height (VH), crypt depth (CD), VH/CD ratio, gut microbiota, blood lipid profile, and thigh meat fatty acid composition.

**Results:**

Dietary inclusion of FCP significantly improved ADWG and LBW, particularly at the 20% level, without affecting ADFI and FCR. Carcass weight and percentage increased significantly (p < 0.05), while abdominal fat and physiological organs remained unaffected. Thymus weight and percentage were significantly increased, indicating enhanced immune response. Intestinal morphology showed increased VH and VH/CD ratio and decreased CD, reflecting improved absorptive capacity. Microbial analysis revealed reduced *Escherichia coli* and increased lactic acid bacteria populations. Blood analysis indicated reduced total cholesterol, triglycerides, and low-density lipoprotein, with increased high-density lipoprotein (p < 0.05). Although meat cholesterol was unchanged, FCP improved fatty acid composition by increasing omega-3, omega-6, omega-9, docosahexaenoic acid and eicosapentaenoic acid levels.

**Conclusion:**

FCP is an effective functional feed ingredient that enhances growth performance, gut health, immune status, and lipid metabolism in broiler chickens. Inclusion up to 20% can be recommended as a sustainable and economically viable strategy to improve poultry productivity and meat quality.

## INTRODUCTION

Fermented feed serves as an economical substitute for traditional feeds in broiler farming, particularly in low-income nations, by utilizing abundant non-conventional feed sources such as cassava peel waste [1]. Notwithstanding obstacles such as elevated cellulose, lignin, and hydrogen cyanide (HCN) concentrations that may hinder poultry nutrition, fermentation enhances feed quality by improving digestibility, increasing protein content, and reducing antinutritional factors such as lignocellulose [2, 3]. It generates bioactive compounds that enhance gut health, immunity, and broiler performance, including growth, carcass quality, and reduced antibiotic use [4, 5]. Fermentation by *Pleurotus ostreatus* markedly decreases fiber content [6].

A blend of 80% cassava peel and 20% tofu dregs exhibits 11.39% crude protein (CP), 22.53% crude fiber, 12.30% lignin, and 11.48% cellulose. Fermenting cassava peel with *Pleurotus ostreatus* for 9 days can increase CP, metabolizable energy (ME), amino acids, and unsaturated fatty acids, while producing secondary metabolites such as 1.80 mg quercetin equivalent (EQ)/100 g flavonoids, 150 mg gallic acid equivalent (GAE)/100 g polyphenols, and 18.5% β-glucans, alongside a reduction in HCN.

*Pleurotus ostreatus* produces bioactive substances such as flavonoids, polyphenols, phenolic compounds, and β-glucans, which influence intestinal structure and health in broilers through antioxidant, anti-inflammatory, prebiotic, and immunomodulatory pathways [7]. Flavonoids function as antimicrobials and antioxidants, safeguarding mucosa, improving nutrient absorption, enhancing microbial diversity, increasing villus height-to-crypt depth ratio (VH/CD), and reducing fat deposition [8–10]. Phenolic compounds serve as prebiotics, improving gut microbiota balance and alleviating oxidative stress [7, 11], whereas polyphenols enhance broiler performance by facilitating absorption, reducing stress, and modulating immune responses [12]. β-glucans enhance microbiota proliferation, nutrient absorption, body mass, feed efficiency, and immune function, potentially reducing antibiotic use [13–15]. Statins such as lovastatin regulate gut immunity and lipid metabolism [16, 17], while fermented feed acts as a prebiotic and antibacterial agent, suppressing pathogens such as *Escherichia coli* and *Salmonella* while promoting lactic acid bacteria (LAB) [14, 18, 19].

Indonesia is the fourth-largest producer of cassava worldwide, with an annual yield of 16.67 million tons. In 2022, West Sumatra contributed 143,330 tons, generating approximately 28,666 tons of peel waste suitable for animal feed [20–22].

Although previous studies using *Lactobacillus plantarum* or *Aspergillus niger* have demonstrated improvements in growth performance, LAB populations, and carcass quality in broilers [23, 24], these studies largely overlook critical physiological and functional parameters, including intestinal morphology, blood lipid profiles, and fatty acid composition in meat. Furthermore, existing research predominantly focuses on production performance while neglecting the functional antioxidant potential of *Pleurotus ostreatus*-derived metabolites, such as flavonoids, polyphenols, β-glucans, and statins, in modulating immune responses, gut microbiota, and lipid metabolism.

In addition, limited attention has been given to the integrated evaluation of sustainable agricultural waste utilization, particularly cassava peel combined with tofu by-products, as a functional feed ingredient with both nutritional and environmental benefits. The role of such fermentation systems in enriching omega-3, omega-6, and omega-9 fatty acids in broiler meat remains insufficiently explored.

Therefore, the present study aims to comprehensively evaluate the effects of *Pleurotus ostreatus*-fermented cassava peel (FCP) as a functional feed ingredient on broiler performance, carcass characteristics, immune organ development, intestinal morphology, microbiota composition, blood lipid profile, and meat quality, including fatty acid enrichment. This study also seeks to establish the potential of FCP as a sustainable and locally available feed resource that can enhance productivity while reducing environmental impact and reliance on antibiotics.

The objectives of this study were to: assess the effects of FCP on growth performance, carcass traits, abdominal fat deposition, physiological organs, and immune organs; evaluate intestinal morphology and microbiota composition; and analyze blood lipid profiles and meat quality parameters, including fatty acid composition.

The hypothesis of this study posits that inclusion of up to 20% FCP in broiler diets improves growth performance, carcass quality, immune organ development, intestinal morphology, beneficial bacterial populations (LAB), high-density lipoprotein (HDL), omega-3, omega-6, and omega-9 fatty acids, docosahexaenoic acid (DHA), and eicosapentaenoic acid (EPA), while reducing pathogenic bacteria (*E. coli*), total cholesterol, low-density lipoprotein (LDL), and triglycerides.

## MATERIALS AND METHODS

### Ethical approval

All experimental procedures involving animals were reviewed and approved by the Animal Care and Use Committee of Universitas Andalas, Padang, Indonesia, under approval number 376/UN.16.2/KEP-FK/2025. The study was conducted in accordance with institutional guidelines for the care and use of animals in research and complied with internationally accepted principles of animal welfare and ethical use of animals in scientific experiments.

The experimental design, housing, feeding, handling, sampling, and slaughter procedures were planned to minimize pain, distress, and unnecessary suffering throughout the study period. Broilers were monitored daily for general health status, behavior, feed and water intake, and any signs of stress or disease. Environmental conditions, including temperature, ventilation, litter management, lighting schedule, and access to feed and water, were maintained at appropriate standards to ensure bird welfare. Vaccination and routine flock health management were implemented according to standard poultry husbandry practices.

At the end of the trial, birds selected for sampling were fasted for the required period and handled carefully to minimize stress before slaughter. All slaughter and sample collection procedures were performed in accordance with local halal regulations and accepted welfare considerations. No unnecessary invasive procedures were performed beyond those required to achieve the scientific objectives of the study.

Because this work involved feeding trials, intestinal sampling, blood collection, organ evaluation, and post-slaughter meat quality assessment in broiler chickens, prior ethical clearance was considered essential and was obtained before the commencement of the experiment.

### Study period and location

This study was conducted from July 2025 to October 2025 at the Poultry Farm of Universitas Andalas. Morphological analysis of the intestine was performed at the Histology Laboratory of the Baso Office Veterinary in Bukittinggi and the Central Laboratory of Universitas Andalas. Intestinal microbiota analysis was performed at the Medicine Laboratory, the Microbiology Laboratory, and the Central Laboratory of Universitas Andalas in Padang, West Sumatra, Indonesia, and at the SIG Laboratory of PT Saraswati in Bogor, West Java, Indonesia.

Broiler blood serum lipid profile analysis was conducted in the Biochemistry Laboratory of the Faculty of Medicine, Universitas Andalas. Omega-3, omega-6, and omega-9 fatty acid analysis was conducted in the GIS Laboratory in Bogor. Broiler thigh meat fat analysis was conducted in the Non-Ruminant Nutrition Laboratory of the Faculty of Animal Science, Universitas Andalas, and thigh meat cholesterol analysis was conducted in the Animal Health Veterinary Laboratory of Politani Payakumbuh.

### Fermentation of cassava peel using *P. ostreatus*

The production of fermented products utilized 1000 g of substrate comprising cassava peel and tofu dregs (80:20). Cassava peels were obtained from traditional food production waste (“Keripik Sanjai”), and tofu dregs were obtained from a tofu factory in Padang city. The cassava peel was diced to a dimension of approximately 0.3–0.5 cm, and the tofu dregs were pressed to remove moisture (substrate moisture content 60%).

The substrate was sterilized in an autoclave (Hirayama HVE-50, Hirayama Manufacturing Corporation, Saitama, Japan) at 121°C for 30 min. Then, 8% *P. ostreatus* inoculum (3.5 × 10^9^ CFU/g) was added based on substrate weight, along with 5 mL of mineral solution (selenium, Mn^2+^, Ca^2+^, and Zn^2+^) for every 100 g of substrate.

The incubation period lasted 9 days at room temperature (28°C–30°C) and 80–85% relative humidity, followed by a 24 h drying period under sunlight. The material was ground to a particle size of 2 mm (crumble form), producing FCP.

### Feed formulation and preparation

FCP was added to the feed to reduce corn and soybean meal usage. The treatment feed consisted of starter feed containing 22% and 3050 kcal/kg, followed by a finisher ration (days 22–35) containing 20% and 3100 kcal/kg, according to [25]. The nutritional and bioactive composition of FCP is presented in Table 1, and the composition and nutrient content of the experimental diets are presented in Table 2.

**Table 1:** Nutritional and bioactive content of fermented cassava peel (FCP).

Nutritional content (%)	FCP
Metabolizable energy (kcal/kg)	2750
Dry matter	89.77
Crude protein	20.95
Crude fiber	14.03
Fat	4.54
Calcium	0.56
Phosphorus (available)	0.2
Glutamic acid	1.02
Methionine	0.02
Methionine + cystine	0.55
Lysine	0.04
Saturated fat	1.41
Unsaturated fat	5.05
Omega-3	6.26
Omega-6	19.05
Omega-9	14.14
β-glucan	18.5
Polyphenols (mg GAE/100 g)	150
Flavonoids (mg EQ/100 g)	1.80
Lovastatin (mg/g)	0.76
Antioxidant	56.07
Hydrogen cyanide (ppm)	91.82
Nitrogen retention	60.50
Crude fiber digestibility	54.60

**Table 2:** Feed formulation and nutrient composition during the experiment.

Feed ingredients (%)	Starter phase (1–21 days)					Finisher phase (22–35 days)				

	FCP0	FCP5	FCP10	FCP15	FCP20	FCP0	FCP5	FCP10	FCP15	FCP20
Corn	61.80	58.55	54.30	50.50	47.00	64.80	61.80	57.55	53.25	49.00
Soybean meal	20.00	18.50	17.50	16.50	15.00	15.50	14.00	13.00	12.00	11.00
Fish meal	14.00	14.00	14.00	14.00	14.00	14.00	14.00	14.00	14.00	14.00
Tapioca flour	1.00	1.00	1.00	0.50	0.50	1.50	1.50	1.50	1.50	1.50
Fermented product	0.00	5.00	10.00	15.00	20.00	0.00	5.00	10.00	15.00	20.00
Coconut oil	1.50	1.75	2.00	2.30	2.30	2.50	2.50	2.75	3.00	3.25
Premix	0.50	0.50	0.50	0.50	0.50	0.50	0.50	0.50	0.50	0.50
Bone meal	1.00	0.50	0.50	0.50	0.50	1.00	0.50	0.50	0.50	0.50
Methionine	0.20	0.20	0.20	0.20	0.20	0.20	0.20	0.20	0.25	0.25
Nutritional content (%)										
ME (kcal/kg)	3007	3109	3009	3114	3010	3110	3014	3105	3002	3101
Crude protein	22.14	20.10	22.09	20.01	22.04	20.06	22.03	20.12	22.00	20.17
Crude fiber	3.61	3.51	4.11	3.97	4.61	4.40	5.13	4.84	5.64	5.27
Fat	4.05	5.02	4.29	5.11	4.43	5.43	4.83	5.74	4.82	6.06
Calcium	1.08	1.06	1.09	1.01	1.09	1.08	1.10	1.16	1.10	1.23
Phosphorus (available)	0.59	0.58	0.58	0.51	0.57	0.51	0.55	0.51	0.54	0.51
Methionine	0.55	0.55	0.54	0.52	0.51	0.55	0.54	0.53	0.57	0.56
Methionine + cystine	0.77	0.79	0.82	0.84	0.86	0.72	0.72	0.71	0.71	0.71
Lysine	1.34	1.23	1.30	1.19	1.26	1.16	1.22	1.12	1.18	1.09
β-glucan	0.00	0.93	1.85	2.78	3.70	0.00	0.93	1.85	2.78	3.70
Polyphenols	0.46	7.93	15.40	22.88	30.35	0.48	7.95	15.42	22.89	30.36
Flavonoids	0.09	0.17	0.26	0.34	0.43	0.08	0.16	0.25	0.33	0.42
Lovastatin	0	0.04	0.08	0.11	0.15	0	0.04	0.08	0.11	0.15

In the starter phase, the ration was formulated with 22% iso-protein and 3050 kcal/kg iso-energy, and in the finisher phase, the ration was formulated with 20% iso-protein and 3100 kcal/kg iso-energy [25]. ME = Metabolizable energy.

FCP (20.95% and 2750 kcal/kg) was used to reduce ground corn (8.75% and 3300 kcal/kg) and soybean meal (51.30% and 2240 kcal/kg) in both starter and finisher phases. Although ground corn and soybean meal decreased, this was compensated by fermented products that improved palatability, nutrient digestibility, and production of secondary metabolites, including flavonoids, polyphenols, β-glucan, and lovastatin.

### Management of broiler maintenance

Two hundred day-old chicks (DOC) of broiler strain Lohmann MB202 (PT Japfa Comfeed, Jakarta, Indonesia) were used. Male and female broiler chicks (2:3) were randomly allocated into five groups, each consisting of four replicates of 10 birds with similar initial body weight (40 ± 1.25 g). The experimental groups consisted of the control diet and diets containing 0%, 5%, 10%, 15%, and 20% FCP. The nutritional composition is presented in Table 2. Birds were housed in 20 cages (10 birds per 1 m² pen) with rice hull litter. The temperature program started at 33°C and was reduced by 2°C weekly, and ventilation was adequate. Lighting was continuous for the first 7 days and then adjusted to a 12 L:12 D cycle. Feed and water were provided *ad libitum*. Vaccination included Marek’s disease vaccine (Gallivac Marek’s HVT Rispens, Merck Animal Health, Rahway, NJ, USA) administered subcutaneously on day 1 and Newcastle disease vaccine (Medivac ND Emulsion, Merck Animal Health) administered intramuscularly on day 7. Bird health status was monitored through behavior, feed intake, water intake, and fecal quality. No abnormal conditions were observed during the experimental period.

### Slaughter procedures and data collection

Broilers were processed according to local halal regulations. Body weight, feed intake, and body weight gain were recorded. FCR was calculated as total FI divided by body weight gain. On day 35, birds were fasted for 12 h and weighed to determine LBW. ADWG and ADFI were calculated. Samples (n = 8) were collected to evaluate carcass, abdominal fat, non-carcass organs (heart, liver, gizzard), and immune organs (spleen, thymus, bursa of Fabricius). Carcass percentage, non-carcass organ percentage, and immune organ percentage were calculated as weight relative to body weight × 100.

### Analysis of intestinal morphology and microbial population

After slaughter, intestines were removed, and 2 cm segments of the duodenum, jejunum, and ileum were fixed in 10% buffered formalin. Samples were embedded in paraffin, sectioned at 5 μm, mounted on slides, and stained with hematoxylin and eosin. VH and CD were measured using ImageJ software (National Institutes of Health, Bethesda, MD, USA) with a trinocular microscope (Zeiss Primostar 3, Carl Zeiss, Oberkochen, Germany). VH/CD was calculated. For microbial analysis, digesta samples were collected in sterile containers. *E. coli* was cultured on MacConkey Broth (Merck KGaA, Darmstadt, Germany), and LAB were cultured on de Man, Rogosa, and Sharpe medium (Merck KGaA). Samples were incubated anaerobically at 38°C (24 h for *E. coli*, 48 h for LAB). Bacterial populations were quantified using the total plate count method and expressed as log_10_ CFU/g [27].

### Analysis of blood serum profile

Blood samples (n = 40) were collected from the carotid artery and jugular vein. Samples were allowed to clot and centrifuged for 20 min at 894 × *g* obtain serum. Total cholesterol, triglycerides, HDL, and LDL were measured using DiaSys kits (DiaSys Diagnostic Systems GmbH, Holzheim, Germany) in a photometric system (Microlab 300, ELITechGroup, Puteaux, France). Enzymatic methods included CHOD-PAP for cholesterol and glycerrol-3- phosphate oxidase (GPO) for triglycerides. Measurements were taken at 546 nm. Thigh meat cholesterol was analyzed using a SpectraLab 752 Pro spectrophotometer (Spectrum Instruments, Shanghai, China), and fat content was determined using the Soxhlet method [28].

### Analysis of thigh meat cholesterol

Cholesterol was analyzed after dehydration and grinding using a reflux method [29], followed by measurement using a SpectraLab 752 Pro spectrophotometer

### Analysis of thigh meat fat and fatty acids

Fat content was determined using the Soxhlet method [28]. Fatty acid composition was analyzed using a gas chromatograph (Shimadzu GC-4CM, Shimadzu Corporation, Kyoto, Japan) equipped with a flame ionization detector and a glass column coated with 5% DEGS. The column temperature was maintained at 180°C, while injector and detector temperatures were set at 270°C. Fatty acids were identified based on retention time.

### Statistical analysis

All data were analyzed using Statistical Package for the Social Sciences version 27.0 (IBM Corp., Armonk, NY, USA) [30]. Differences among treatments were evaluated using Duncan’s multiple range test. Results were considered significant at p < 0.05. Data are presented as mean ± standard deviation.

## RESULTS

### Growth performance

The data growth performance of parameters was encapsulated in Table 3. LBW, ADWG, ADFI, and FCR at 35 days of age during the starter and finisher phases indicated that including up to 20% FCP in the diet significantly increased LBW and ADWG (p < 0.05), but ADFI and FCR remained unaffected (p > 0.05). During the starter phase, ADWG ranged from 41.94 to 44.68 g, ADFI ranged from 58.24 to 60.24 g, and FCR ranged from 1.31 to 1.43, with no significant differences across treatments. In the finisher phase, ADWG climbed from 75.13 to 82.77 g, ADFI rose from 129.30 to 137.43 g, and FCR shifted from 1.72 to 1.66. During the 1–35 day phase, body weight increased by 7.03% from 2086.65 g (control) to 2233.40 g (FCP20), ADWG increased from 67.52 to 72.81 g, ADFI rose from 111.54 to 118.13 g, and FCR decreased from 1.65 to 1.62. In summary, FCP supplementation markedly improved LBW and ADWG without influencing feed conversion or intake.

**Table 3:** Growth performance of broilers fed fermented cassava peel (FCP).

Parameter	FCP0	FCP5	FCP10	FCP15	FCP20	SE	p-value
Starter phase							
ADWG, g	44.68 ± 2.14	44.12 ± 2.51	44.64 ± 1.21	41.94 ± 3.00	42.96 ± 3.44	1.29	0.513
ADFI, g	58.24 ± 1.38	59.49 ± 0.38	58.83 ± 1.53	59.70 ± 0.79	60.24 ± 0.25	0.50	0.100
FCR	1.31 ± 0.06	1.35 ± 0.08	1.32 ± 0.03	1.43 ± 0.11	1.41 ± 0.12	0.04	0.301
Finisher phase							
ADWG, g	75.13 ± 2.31ᵇ	76.76 ± 4.27ᵇ	77.14 ± 1.08ᵇ	78.86 ± 0.91ᵇ	82.77 ± 1.95ᵃ	1.29	0.031
ADFI, g	129.30 ± 3.37	131.15 ± 5.74	131.81 ± 3.91	132.30 ± 2.95	137.43 ± 3.12	1.96	0.133
FCR	1.72 ± 0.05	1.71 ± 0.03	1.71 ± 0.03	1.68 ± 0.03	1.66 ± 0.01	0.02	0.952
1–35 days							
LBW, g	2086.65 ± 10.93ᶜ	2114.55 ± 27.60ᵇᶜ	2128.16 ± 20.48ᵇ	2145.82 ± 19.84ᵇ	2233.40 ± 22.35ᵃ	10.47	0.028
ADWG, g	67.52 ± 1.98ᵇ	68.60 ± 3.85ᵇ	69.02 ± 0.77ᵇ	69.63 ± 0.76ᵃᵇ	72.81 ± 2.15ᵃ	1.11	0.041
ADFI, g	111.54 ± 2.68	113.24 ± 4.39	113.56 ± 2.91	114.15 ± 2.07	118.13 ± 2.39	1.50	0.107
FCR	1.65 ± 0.03	1.65 ± 0.03	1.65 ± 0.02	1.64 ± 0.03	1.62 ± 0.02	0.01	0.956

ᵃ–ᶜValues within a row with different superscripts differ significantly (p < 0.05) for ADWG in the finisher phase, LBW, and ADWG during 1–35 days. ns = non-significant (p > 0.05). ADWG = Average Daily Weight Gain, ADFI = Average Daily Feed Intake, FCR = Feed Conversion Ratio, LBW = Live Body Weight.

### Carcass parameters and physiological organs

Carcass parameters and physiological organs are presented in Table 4. The carcass weight was highly significant (p < 0.01), increasing from 1465.50 g per animal in the FCP0 treatment to 1628.00 g per animal in the FCP20 treatment. The carcass percentage significantly increased (p < 0.05) from 70.23% in the control group to 72.12% in the FCP15 group and 72.90% in the FCP20 group. The inclusion of FCP in the diet did not influence (p > 0.05) the weight and proportion of physiological organs (liver, heart, and gizzard).

**Table 4:** Carcass parameters and physiological organs.

Parameter	FCP0	FCP5	FCP10	FCP15	FCP20	SE	p-value
Carcass, g	1465.50 ± 16.58ᵈ	1495.00 ± 27.80ᶜᵈ	1522.00 ± 17.03ᵇᶜ	1547.50 ± 14.79ᵇ	1628.00 ± 8.60ᵃ	10.43	0.001
Abdominal fat, g	34.00 ± 2.00	35.00 ± 1.41	34.00 ± 2.71	35.25 ± 0.96	35.50 ± 3.00	1.08	0.785
Carcass, %	70.23 ± 0.65ᶜ	70.70 ± 1.04ᵇᶜ	71.53 ± 1.52ᵃᵇᶜ	72.12 ± 0.58ᵃᵇ	72.90 ± 1.13ᵃ	0.52	0.041
Abdominal fat, %	1.63 ± 0.09	1.66 ± 0.08	1.60 ± 0.13	1.64 ± 0.04	1.59 ± 0.15	0.05	0.888
Liver, g	33.75 ± 2.50	35.50 ± 3.79	32.00 ± 4.69	35.50 ± 2.52	39.00 ± 5.77	2.03	0.216
Heart, g	10.00 ± 1.41	9.00 ± 2.16	9.50 ± 1.29	8.50 ± 0.58	9.75 ± 1.71	0.76	0.651
Gizzard, g	32.55 ± 3.20	35.50 ± 4.73	32.50 ± 6.14	35.00 ± 5.83	35.00 ± 2.94	2.38	0.790
Liver, %	1.62 ± 0.12	1.68 ± 0.19	1.50 ± 0.22	1.65 ± 0.11	1.75 ± 0.26	0.09	0.543
Heart, %	0.48 ± 0.07	0.43 ± 0.11	0.45 ± 0.06	0.40 ± 0.03	0.44 ± 0.08	0.04	0.607
Gizzard, %	1.55 ± 0.15	1.68 ± 0.24	1.53 ± 0.29	1.63 ± 0.26	1.57 ± 0.13	0.11	0.845

ᵃ–ᵈValues within a row with different superscripts differ highly significantly (p < 0.01) for carcass weight (g). ᵃ–ᶜValues within a row with different superscripts differ significantly (p < 0.05) for carcass percentage. ns = non-significant (p > 0.05).

### Immune system organs

Data on the immunological organs were presented in Table 5. The effect of FCP was highly significant (p < 0.01) on thymus weight but significant (p < 0.05) on thymus percentage. The thymus weight rose from 1.50 g per bird in the control group to 3.13 g per bird in the FCP20. The thymus percentage rose from 0.07% in the control treatment to 0.14% in the FCP20. The administration of FCP did not significantly affect (p > 0.05) the weight and percentage of the bursa of Fabricius and spleen. This suggests that FCP treatment can promote thymus development without influencing other lymphoid organs.

**Table 5:** Immune organ parameters.

Parameters measured	FCP0	FCP5	FCP10	FCP15	FCP20	SE	p-value
Bursa of Fabricius, g	4.75 ± 0.96	4.25 ± 1.26	4.75 ± 0.96	4.25 ± 0.50	5.25 ± 2.22	0.66	0.800
Spleen, g	1.75 ± 0.50	2.50 ± 0.58	2.00 ± 0.82	2.25 ± 0.50	2.50 ± 1.00	0.35	0.515
Thymus, g	1.50 ± 0.58ᵇ	1.75 ± 0.50ᵇ	1.75 ± 0.96ᵇ	2.25 ± 0.50ᵃᵇ	3.13 ± 0.25ᵃ	0.30	0.001
Bursa of Fabricius, %	0.23 ± 0.05	0.20 ± 0.06	0.22 ± 0.04	0.20 ± 0.02	0.24 ± 0.10	0.03	0.878
Spleen, %	0.08 ± 0.02	0.12 ± 0.03	0.09 ± 0.04	0.10 ± 0.02	0.11 ± 0.04	0.02	0.585
Thymus, %	0.07 ± 0.03ᶜ	0.08 ± 0.02ᵇᶜ	0.08 ± 0.04ᵇᶜ	0.10 ± 0.02ᵃᵇ	0.14 ± 0.01ᵃ	0.01	0.035

ᵃ,ᵇValues within a row with different superscripts differ highly significantly (p < 0.01) for thymus weight (g). ᵃ–ᶜValues within a row with different superscripts differ significantly (p < 0.05) for thymus percentage. ns = non-significant (p > 0.05).

### Intestinal morphology

**Figure 1:**
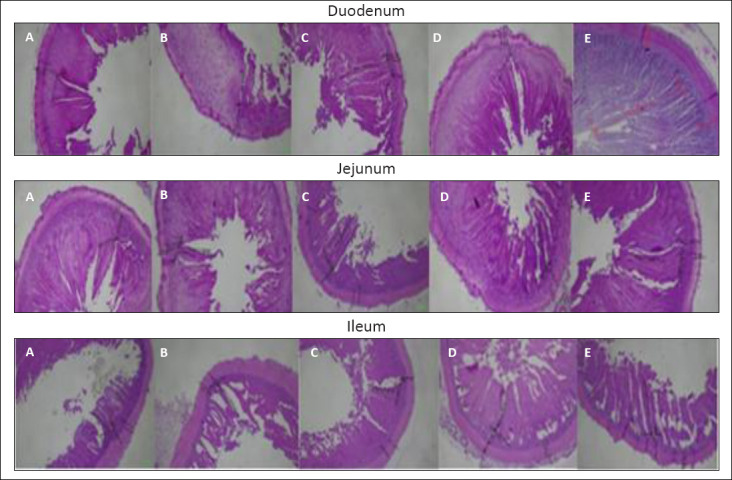
Histological sections of the small intestine (duodenum, jejunum, and ileum) of broilers treated with fermented cassava peel (FCP). The top row represents the duodenum, the middle row represents the jejunum, and the bottom row represents the ileum. (A) Control group without FCP treatment (FCP0), (B) FCP5, (C) FCP10, (D) FCP15, and (E) FCP20. Sections were stained with hematoxylin and eosin. Scale bar = 200 µm.

**Figure 2:**
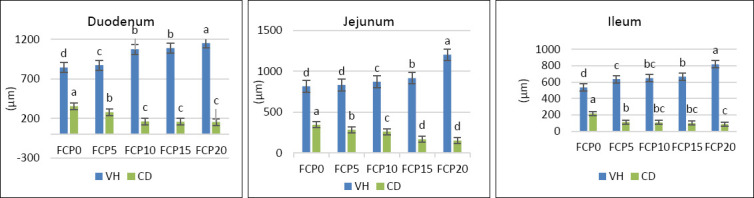
Villus height (VH) and crypt depth (CD) in the small intestine (duodenum, jejunum, and ileum). Different letters above the bars indicate highly significant differences among treatment groups (p < 0.01), whereas significant differences (p < 0.05) were observed for duodenal VH and ileal CD. The green bars represent VH, and the blue bars represent CD.

Figure 1 and Figure 2 illustrates the intestinal morphology of the duodenum, jejunum, and ileum. Intestinal morphology results showed that duodenal VH significantly increased (p < 0.05) in broilers administered only in FCP20, while jejunal and ileal VH highly significantly increased (p < 0.01) in FCP20-treated broilers (Table 6). In contrast, CD was increased in FCP0. The VH/CD ratio in the duodenum was highly significantly elevated (p < 0.01) across the FCP10, FCP15, and FCP20 groups, but in the jejunum and ileum, it increased significantly only in the FCP20 group. These changes indicate an expanded surface area for nutrient absorption, with higher VH/CD ratios potentially reflecting improved digestive and absorptive functions in the broiler small intestine.

**Table 6:** Intestinal morphology.

Parameters	FCP0	FCP5	FCP10	FCP15	FCP20	SE	p-value
Duodenum							
VH	844.96 ± 14.43ᵈ	873.26 ± 17.32ᶜ	1075.05 ± 13.90ᵇ	1090.43 ± 14.90ᵇ	1152.36 ± 10.83ᵃ	7.21	0.001
CD	354.90 ± 4.37ᵃ	280.89 ± 1.73ᵇ	162.21 ± 33.27ᶜ	157.65 ± 9.12ᶜ	154.66 ± 2.94ᶜ	7.81	0.031
VH/CD	2.38 ± 0.03ᵇ	3.11 ± 0.08ᵇ	6.90 ± 1.76ᵃ	6.93 ± 0.42ᵃ	7.45 ± 0.09ᵃ	0.40	0.001
Jejunum							
VH	815.61 ± 9.56ᵈ	831.88 ± 16.91ᵈ	870.98 ± 12.86ᶜ	916.23 ± 9.51ᵇ	1202.92 ± 8.30ᵃ	5.91	0.001
CD	343.62 ± 6.52ᵃ	282.88 ± 13.09ᵇ	259.12 ± 5.36ᶜ	166.43 ± 15.26ᵈ	150.40 ± 11.44ᵈ	5.51	0.001
VH/CD	2.55 ± 0.32ᶜ	2.95 ± 0.14ᶜ	3.36 ± 0.10ᶜ	5.54 ± 0.45ᵇ	8.03 ± 0.06ᵃ	0.19	0.001
Ileum							
VH	534.89 ± 5.49ᵈ	635.82 ± 9.27ᶜ	648.34 ± 12.15ᵇᶜ	668.14 ± 23.69ᵇ	816.90 ± 8.17ᵃ	6.68	0.001
CD	218.09 ± 15.04ᵃ	113.07 ± 1.73ᵇ	106.89 ± 13.54ᵇ	99.42 ± 16.04ᵇᶜ	85.75 ± 7.69ᶜ	7.27	0.024
VH/CD	2.46 ± 0.14ᵈ	5.76 ± 1.12ᶜ	6.13 ± 0.67ᵇᶜ	6.86 ± 1.17ᵇ	9.58 ± 0.85ᵃ	0.44	0.001

ᵃ–ᵈValues within a row with different superscripts differ highly significantly (p < 0.01). ᵃ–ᵈValues within a row with different superscripts differ significantly (p < 0.05) for duodenal VH and ileal CD. VH = Villi Height, CD = Crypt Depth, VH/CD = Ratio of VH to CD.

### Intestinal microbial population

**Figure 3:**
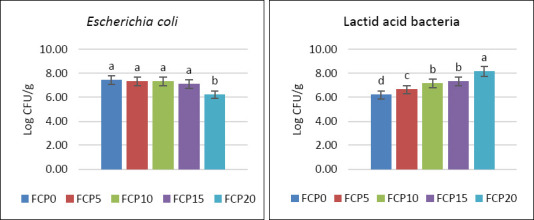
Microbial population of pathogenic bacteria (*Escherichia coli*) and lactic acid bacteria in the small intestine of broilers. Different letters above the bars indicate highly significant differences among treatment groups (p < 0.01).

The total population of *E. coli* (Table 7 and Figure 3) showed significant differences across treatments (p < 0.01), with FCP20 exhibiting a substantial reduction (p < 0.01), and FCP0 reaching the lowest count of 6.23 log CFU/g, indicating that increasing the fermentation level of *P. ostreatus* diminishes *E. coli* populations in broiler intestines. Similarly, LAB population differed significantly among treatments (p < 0.01), rising progressively from FCP5 to FCP10 and FCP15, and peaking at FCP20, with a significant increase (p < 0.01) to 8.14 log CFU/g, demonstrating that higher fermentation levels of *P. ostreatus* markedly elevate beneficial LAB populations in broiler intestines.

**Table 7:** Intestinal microbial population.

Parameters (log CFU/g)	FCP0	FCP5	FCP10	FCP15	FCP20	SE	p-value
*Escherichia coli*	7.44 ± 0.02ᵃ	7.34 ± 0.01ᵃ	7.33 ± 0.02ᵃ	7.10 ± 0.48ᵃ	6.23 ± 0.03ᵇ	0.12	0.001
Lactic acid bacteria	6.19 ± 0.07ᵈ	6.65 ± 0.48ᶜ	7.17 ± 0.07ᵇ	7.35 ± 0.03ᵇ	8.14 ± 0.05ᵃ	0.13	0.001

ᵃ–ᵈValues within a row with different superscripts differ highly significantly (p < 0.01).

### Blood and meat cholesterol

Table 8 summarizes cholesterol levels in meat and blood. The administration of fermented products containing *P. ostreatus* reduced total cholesterol, triglycerides, and LDL levels (p < 0.01) and increased HDL levels (p < 0.01) in broilers. During the FCP5, FCP10, FCP15, and FCP20 treatments, total blood cholesterol levels decreased, reaching their lowest at FCP20 (112.20 ± 2.60 mg/dL). LDL values decreased in FCP10, FCP15, and FCP20; HDL levels increased in FCP15 and FCP20 (lowest in FCP0). Total protein ranged from 5.51 ± 0.73 to 6.10 ± 0.32 g/dL, and glucose ranged from 10.80 ± 0.39 to 12.40 ± 1.46 mmol/dL. Increasing the fermented feed content to 10–20% improved the blood lipid profile by decreasing LDL and total cholesterol, increasing HDL at FCP15–20%, and decreasing triglycerides at FCP20.

**Table 8:** Blood lipid profile.

Parameters measured	FCP0	FCP5	FCP10	FCP15	FCP20	SE	p-value
Total cholesterol (mg/dL)	151.63 ± 25.10ᵃ	133.63 ± 20.60ᵃᵇ	120.60 ± 15.5ᵇ	118.78 ± 19.40ᵇ	114.65 ± 2.60ᵇ	9.20	0.036
Triglycerides (mg/dL)	29.00 ± 2.4ᵃ	28.45 ± 6.00ᵃ	26.35 ± 5.30ᵃ	24.43 ± 4.30ᵃ	17.18 ± 1.70ᵇ	2.10	0.040
HDL (mg/dL)	43.38 ± 4.95ᵇ	46.20 ± 5.16ᵇ	46.33 ± 2.76ᵇ	51.13 ± 7.29ᵃᵇ	54.63 ± 0.76ᵃ	2.37	0.031
LDL (mg/dL)	102.43 ± 23.53ᵃ	81.70 ± 18.19ᵃᵇ	68.95 ± 11.76ᵇ	62.70 ± 15.06ᵇ	55.85 ± 18.39ᵇ	8.91	0.022
Total protein (g/dL), ns	5.51 ± 0.73	5.74 ± 0.65	5.89 ± 0.55	6.00 ± 0.63	6.10 ± 0.32	0.30	0.062
Glucose (mmol/dL), ns	12.40 ± 1.46	11.90 ± 1.41	11.40 ± 1.22	11.00 ± 0.34	10.80 ± 0.39	0.54	0.084
Thigh meat cholesterol (mg/100 g)	204.80 ± 0.25	204.35 ± 0.94	203.69 ± 0.47	203.87 ± 0.64	203.90 ± 0.28	0.29	0.612

ᵃ^,^ᵇValues within a row with different superscripts differ highly significantly (p < 0.01). ns = non-significant (p > 0.05).

Treatment FCP0 to FCP20 did not significantly alter the cholesterol content of roasted thigh meat (p > 0.05), indicating that this intervention had little effect on the cholesterol content of the meat.

### Thigh meat fat and fatty acids

Table 9 displays the fat and fatty acid content of thigh meat. Although the fat content of roasted thigh meat varied across treatments, no consistent pattern emerged with increasing amounts of fermented products, ranging from 6.75% (FCP0) to 7.69% (FCP20).

**Table 9:** Fatty acid profile of thigh meat.

Parameters mg/100 g	FCP0	FCP5	FCP10	FCP15	FCP20	SE	p-value
Omega-3	290.40 ± 6.50ᵉ	322.40 ± 5.89ᵈ	449.60 ± 2.86ᶜ	535.70 ± 3.78ᵇ	594.15 ± 3.96ᵃ	2.40	0.001
Omega-6	3275.30 ± 6.99ᵉ	3447.35 ± 6.96ᵈ	3613.50 ± 8.20ᶜ	3976.75 ± 8.09ᵇ	4253.65 ± 6.13ᵃ	3.66	0.001
Omega-9	6215.60 ± 6.25ᵈ	6653.05 ± 3.87ᶜ	6948.10 ± 6.14ᶜ	7280.85 ± 4.49ᵇ	7656.80 ± 5.16ᵃ	2.63	0.001
DHA	176.70 ± 6.61ᵈ	168.95 ± 5.46ᵈ	309.90 ± 4.82ᶜ	407.40 ± 7.09ᵇ	466.40 ± 6.52ᵃ	3.08	0.001
EPA	24.75 ± 6.59ᵇ	28.10 ± 6.81ᵇ	46.55 ± 4.49ᵃ	47.65 ± 7.61ᵃ	52.45 ± 6.55ᵃ	3.25	0.001
Omega-6/omega-3	11.28 ± 0.26ᵉ	10.70 ± 0.19ᵈ	8.04 ± 0.05ᶜ	7.42 ± 0.04ᵇ	7.16 ± 0.04ᵃ	0.07	0.032
SFA (%)	28.48ᵃ	26.99ᵃ	25.35ᵇ	24.00ᵇᶜ	23.48ᶜ	0.54	0.033
MUFA (%)	42.50ᵇ	43.28ᵃᵇ	44.63ᵃ	45.10ᵃ	45.50ᵃ	0.48	0.034
PUFA (%)	25.02ᵇ	25.73ᵇᶜ	26.02ᵇ	26.90ᵃ	27.02ᵃ	0.28	0.031
SFA/MUFA + PUFA (%)	1:2.37	1:2.55	1:2.78	1:3.00	1:3.08		
Thigh meat fat (%), ns	6.75	8.16	8.20	7.64	7.69	2.34	0.663

ᵃ–ᵉValues within a row with different superscripts differ significantly (p < 0.05)

The fatty acid composition in roasted thigh meat markedly elevates the levels of unsaturated fatty acids, evidenced by an increase at FCP0 to FCP20 in omega-3 content from 290.40 ± 6.50 to 594.15 ± 3.96 mg/100 g, omega-6 content from 3275.30 ± 6.99 to 4253.65 ± 6.13 mg/100 g, and omega-9 content from 6215.60 ± 6.25 to 7656.80 ± 5.16 mg/100 g, DHA 176.70 ± 6.61 to 466.40 ± 6.52 mg/100 g, EPA 24.75 ± 6.59 to 52.45 ± 6.55 mg/100 g, ratio omega-6/omega-3 ranged from 7.16 ± 0.04 to 11.28 ± 0.26, SFA 23.48 to 28.48%, MUFA 42.50 to 45.50%, PUFA 25.02 to 27.02%, ratio SFA/MUFA + PUFA 1:2.37 to 1:3.08, and thigh meat fat 6.75 to 7.69%.

## DISCUSSION

### Growth performance

Fermented products containing *P. ostreatus* enhance broiler performance, particularly in ADWG finisher phase and ADWG and LBW at 1–35 days, in the FCP20 treatment compared to the control group, due to the fermentation process with *P. ostreatus*, which improves the nutritional quality of feed ingredients, particularly through its ability to produce enzymes such as cellulase and ligninase that break down complex compounds into simpler forms that are easily absorbed by the broiler digestive system. Studies show the fungus boosts the nutritional quality of agricultural waste by elevating energy, CP, microbial biomass, and volatile fatty acids [30, 31], transforms waste into nutritious feed via cellulose digestion [32, 33], increases intake and digestibility of dry matter, protein, organic matter, fiber, and energy [31, 34], delignifies substrates (reducing lignin by up to 46.9% and raising protein by 69.8%) [35], and produces aromas/flavors that stimulate appetite and optimize nutrient utilization [36].

FCP was given to reduce ground corn and soybean meal usage in feed, both in the starter and finisher phases. Although ground corn and soybean meal were reduced, this was compensated for by fermented products that increased palatability and nutrient digestibility and produced secondary metabolites. Fermentation with *P. ostreatus* produces bioactive compounds, including flavonoids, polyphenols, and β-glucans. Bioactive compound produced from FCP contained 1.80 mg EQ/100 g flavonoids, 150 mg GAE/100 g polyphenols, and 18.5% β-glucans. The increase in flavonoid (0.43 mg EQ/100 g), polyphenol (30.35 mg GAE/100 g), and β-glucan (3.7%) in the starter phase, and flavonoid (0.42 mg EQ/100 g), polyphenol (30.36 mg GAE/100 g), and β-glucan (3.7%) in the finisher phase in FCP20. Elevated concentrations of β-glucans, flavonoids, and polyphenols in broiler feed can enhance intestinal health, mitigate oxidative stress, and bolster the immune system, thereby facilitating the allocation of energy and nutrients toward body growth, ultimately resulting in increased ADWG and LBW. Flavonoids protect cells from oxidative stress, strengthen the immune system, and maintain digestive health [31]. β-glucan acts as an immunostimulant, increasing macrophage activity and improving antibody responses [14]. Polyphenol extracts from fermented cereal-based feed can reduce corticosterone levels and improve production performance, including increased body weight [37].

Specific results showed a 7.03% increase in LBW and a 10.15% increase in ADWG with fermented cassava peel in FCP20. A 12% increase in ADWG was reported [38], and a 14% increase in body weight was noted [39], both of which were also increased with fermented wheat bran by *P. ostreatus*.

### Carcass and physiological organs

Fermented products containing *P. ostreatus* improve broiler carcass performance by highly significantly (p < 0.01) increasing carcass weight and significantly increasing percentage (p < 0.05), attributed to enhanced digestibility, nutrient availability, muscle protein deposition, and feed efficiency. FCP products are easier to digest because *P. ostreatus* has the ability to break down crude fiber and antinutrients into simpler forms through fermentation, resulting in more efficient protein and energy absorption. Enzymes such as cellulase and ligninase from *P. ostreatus* also accelerate digestion and nutrient absorption, thereby supporting optimal growth and carcass formation. Also, the FCP20 product produces higher levels of bioactive chemicals, which can improve average daily weight gain (ADWG) and increase carcass weight.

In line with previous results, the fermentation process using *P. ostreatus* can convert waste such as cassava peel and tofu pulp into high-value feed by degrading cellulose, thereby increasing the intake and digestibility of dry matter, protein, organic matter, fiber, and energy [30, 31]. In addition, the enzymatic activity of this fungus reduces lignin content by up to 46.9% and increases protein content by up to 69.8%, thereby improving digestibility and nutrient utilization efficiency in fermented feed [31, 34]. Supporting studies reported higher carcass weight and percentage with fermented feed [39, 40], and improvements in carcass traits have also been observed [38].

This study found no significant effects of fermented products containing *P. ostreatus* on abdominal fat, liver, heart, or gizzard weights/percentages (p > 0.05). This was due to a reduction in the crude fiber content of cassava peel after fermentation with *P. ostreatus*, which resulted in a lighter workload on the digestive system and fiber metabolism; thus, the changes in these organs were not large enough to be biologically or statistically significant. The use of FCP in poultry diets does not significantly alter the relative weights of the heart, liver, and gizzard, suggesting that the improved fiber quality and digestibility do not induce abnormal physiological enlargement of these internal organs [41]. No impacts on visceral organs by feeding fermented rice bran have also been reported [39].

### Immune system organs

Fermented products containing *P. ostreatus* also boost immunological organs, notably raising thymus gland weight and percentage via β-glucan components, with no significant effects on the bursa of Fabricius or spleen. Increased β-glucan content in FCP15 (2.78%) and FCP20 (3.70%) can enhance the thymus gland. β-glucan interacts with receptors on macrophages and intestinal cells, subsequently transmitting signals to lymphoid and peripheral organs, including the thymus. β-glucan enhances phagocytic activity and nitric oxide synthesis, which stimulates T lymphocyte differentiation and proliferation, strengthens macrophage phagocytic activity, and optimizes the cellular immune response [42]. In this study, without causing excessive hypertrophy of the bursa of Fabricius or the spleen, only the thymus shows increased weight and percentage, while the other two organs remain within the normal physiological range. Increased thymus weight and immune response enhancement without affecting other lymphoid organs have been reported [43, 44].

### Intestinal morphology

Table 6 shows that the highest villi value (VH) and the lowest CD value are achieved in treatment FCP20, indicating that FCP20 can improve intestinal absorption capacity as a functional feed by stimulating VH growth in the duodenum, jejunum, and ileum. In treatment FCP20, the villi value is high, and the CD is low, likely due to fermentation by *P. ostreatus* and its bioactive compounds. *P. ostreatus* produces bioactive compounds, including flavonoids, polyphenols, and β-glucans, that improve intestinal structure through antioxidant, anti-inflammatory, prebiotic, and immunomodulatory effects [7]. FCP20 enhances VH and VH/CD ratio potentially via β-glucan, polyphenols, flavonoids, and antioxidants. Polyphenols, flavonoids, and antioxidants improve nutrient absorption, as evidenced by elongated VH and VH/CD ratio and reduced CD [45].

Intestinal villi are the primary structures for nutritional absorption, with higher total villi (VH) enhancing absorption capacity. The jejunum exhibits the highest density of villi compared to the duodenum and ileum, signifying excellent nutrient absorption in this segment. Treatment FP0 was considered inferior due to its anti-nutrient content, including lectins from ground corn and soybean meal, which may lead to chronic inflammation, villi atrophy, and an increase in pathogenic bacteria, ultimately resulting in shorter villi. Research indicates substantial improvements in this ratio with FCP, reaching a peak at FCP20, accompanied by morphometric advancement from the duodenum to the ileum, suggesting prolonged nutrient absorption associated with gut health and growth efficacy [46]. These alterations mirror the effects of fermented foods such as rapeseed and soybean, which enhance villus development via lactic acid and metabolites [47].

Subsequent analysis reveals a notable trend: CD decreases with increasing FCP doses, especially in the FCP20 treatment, which shows the lowest CD across all intestinal segments. The ramifications for gut health resulting from this reduction in CD are significant in broiler production, where shallow crypts indicate an ideal balance between cellular regeneration and villus development. Increases in VH and VH/CD were associated with improved nutrient absorption and growth efficiency, further corroborated by *P. ostreatus* compounds that alter villus morphology to enhance intestinal health and absorption in broilers, consistent with research on the relationship between VH and gut health in antibiotic scenarios [48]. A heightened VH/CD ratio indicates an increased absorptive surface area and enhanced digestion capacity [49].

### Intestinal microbial population

According to Table 7, the improvement in the small intestinal bacterial population in the FCP20 treatment group was associated with a decrease in the population of harmful bacteria (*E. coli*) and an increase in LAB. Flavonoids, polyphenols, and β-glucans exhibit a distinctive capacity to inhibit pathogenic bacteria such as *E. coli* while concurrently enhancing the proliferation of *Lactobacillus acidophilus*. The dual mechanisms of selective antibacterial action and prebiotic effects render flavonoids and polyphenols essential for preserving gut health. Bioactive compounds, including β-glucans and secondary metabolites from *P. ostreatus*, contribute to this selective antimicrobial effect by adhering to the cell walls of pathogenic bacteria while preserving probiotic bacteria, thus sustaining a balanced gut microbiota ecosystem [50]. Polyphenols elevate *Lactobacillus*/ *Bifidobacterium* levels and inhibit pathogens, thereby improving villus morphology and nutrient absorption, which correlates with a diminished intestinal pathogen load and lower serum globulin levels [51, 52]. The fermentation products of *P. ostreatus*, especially the stem residue, enhance LAB and reduce *E. coli*/*Salmonella* in broilers, likely owing to the antimicrobial and supportive characteristics of polysaccharides, β-glucans, peptides, phenolic compounds, and terpenoids [53]. These products suppress pathogen proliferation and enhance microbial equilibrium [53, 54], whereas oyster mushroom extracts exhibit potent antibacterial activity against *E. coli* and other pathogens and concurrently augment antioxidant capacity [55, 56].

### Blood and thigh meat cholesterol

Broilers fed a fermented product containing *P. ostreatus* showed decreases in blood total cholesterol, triglycerides, and LDL, but an increase in HDL (p < 0.01). The FCP20 therapy decreased total cholesterol by 26%, triglycerides by 40.76%, LDL by 45.47%, and increased HDL by 25.93%, but did not differ in total protein or glucose compared to the control. The increased HDL and decreased total cholesterol, triglycerides, and LDL were linked to enhanced digestion of nutrients and bioactive compounds, including β-glucan, lovastatin, flavonoid, and polyphenols (highest in FCP20), which act as antioxidants and enhance lipid metabolism. β-glucans and statins function as immunomodulators and antihypercholesterolemic agents. The combination of statins (lovastatin) with β-glucan from mushrooms reduces total and LDL cholesterol in broilers by up to 30% due to synergistic inhibition of HMG-CoA reductase and lipid binding [57]. Supplementation with oyster mushrooms has demonstrated a reduction in total cholesterol, triglycerides, LDL, and very-LDL in cockerel chickens, while increasing HDL [58].

The meat’s cholesterol content remained unchanged, despite a drop in blood cholesterol. Cholesterol in meat is a structural component of the animal’s muscle cell membranes. This cholesterol is relatively stable and cannot be removed simply by surface fermentation or by adding fungi after slaughter. Fungal active substances cannot effectively penetrate and degrade cholesterol that is already bound in dead muscle tissue by biological or chemical mechanisms [59, 60].

### Thigh meat fat, and fatty acid

The fat thigh meat is not affected by the application of fermented products containing *P. ostreatus*. The meat’s fat content remained unchanged because the meat cholesterol did not change. Administering fermented products containing *P. ostreatus* to broilers significantly enhanced the fatty acid profile of thigh meat, with the FCP20 treatment increasing omega-3, omega-6 (PUFA), omega-9 (MUFA), DHA, and EPA compared to the control (p > 0.05). This improvement is linked to FCP20, which has high nutrient digestibility, and to bioactive compounds such as β-glucan, polyphenols, and flavonoids, which offer antioxidant and antimicrobial properties. β-glucans boost nutrient bioavailability by degrading cellulose, thereby reducing lipid oxidation and enhancing metabolic health, which improves meat composition [60]. β-glucans promote PUFA biosynthesis by elevating enzyme activity, while flavonoids and polyphenols prevent fatty acid degradation during storage, preserving a healthy profile that supports reduced inflammation and improved meat quality for human health. Comparative studies reported a 22% PUFA increase from polyphenols [60], a 21% rise via β-glucans and polyphenols [61], and an improved omega-6/omega-3 ratio through fungal antioxidants [62].

The fatty acid profile in meat is more easily modifiable by diet than meat cholesterol levels, which are more stable due to regulatory mechanisms governing cholesterol synthesis and distribution in the animal’s body [63]. This disparity illustrates the overarching metabolic regulation in animals: fatty acid accumulation is significantly affected by dietary intake, whereas cholesterol concentrations are regulated by homeostatic mechanisms. Numerous studies indicate that the fatty acid composition of meat varies by species and feeding methods, with nutritional approaches such as grass-feeding or omega-3 supplementation increasing PUFA levels and optimizing the omega-6/omega-3 ratio [62]. The increased intake of unsaturated fatty acids, particularly omega-3 and omega-6, confers significant health benefits, particularly when they replace saturated fatty acids in the diet. Omega-3 fatty acids, including EPA and DHA, are crucial for cardiovascular protection, modulation of inflammation, and brain function [63].

The fermentation study of cassava peel utilizing *L*. *plantarum* or *A. niger* demonstrated enhanced growth, elevated LAB concentration, and improved carcass quality; however, the metabolic compounds generated remain unexamined, and the impact of fermentation on intestinal morphology, cholesterol levels, and fatty acid composition in the meat has yet to be assessed. This study demonstrated that the fermentation of cassava peel and *P. ostreatus* enhanced nutritional value and digestibility while generating bioactive chemicals that influence performance, immune organ function, intestinal shape, gut microbiota composition, cholesterol levels, and fatty acid profiles. Incorporating up to 20% FCP products into broiler feed is economically advantageous because it leverages agricultural waste, thereby reducing the need for costly ground maize (23.95–24.38%) and soybean meal (25–29.03%), ultimately benefiting farmers in Indonesia.

## CONCLUSION

The present study demonstrated that dietary inclusion of FCP, particularly at 20%, significantly improved growth performance, as evidenced by increased LBW and ADWG, without adversely affecting ADFI or FCR. Carcass traits were enhanced, with higher carcass weight and percentage, while physiological organs remained unaffected, indicating no detrimental impact on organ development. Immune response was positively modulated through increased thymus weight and percentage, suggesting improved cellular immunity. Intestinal health was markedly improved, as reflected by increased VH, reduced CD, and a higher VH/CD ratio, alongside a favorable shift in gut microbiota characterized by reduced *E. coli* and increased LAB populations. Furthermore, FCP supplementation improved blood lipid profiles by decreasing total cholesterol, triglycerides, and LDL, while increasing HDL. Although meat cholesterol remained unchanged, the fatty acid composition of thigh meat improved significantly, with increased omega-3, omega-6, omega-9, DHA, and EPA levels, enhancing the nutritional quality of broiler meat.

From a practical perspective, the use of FCP offers a sustainable and cost-effective feeding strategy by utilizing agricultural waste, thereby reducing dependence on conventional feed ingredients such as maize and soybean meal. This approach not only lowers production costs but also contributes to environmental sustainability through waste valorization. The improved growth performance, carcass yield, gut health, and lipid profile collectively indicate that FCP can serve as a functional feed ingredient in broiler production systems.

A key strength of this study lies in its comprehensive evaluation of FCP, encompassing growth performance, carcass traits, immune response, intestinal morphology, microbial population, and lipid metabolism. This integrated approach provides a holistic understanding of the biological effects of FCP in broilers. Additionally, the use of a graded inclusion level allows for clear identification of the optimal dietary level.

However, certain limitations should be acknowledged. The study was conducted under controlled experimental conditions, which may not fully reflect commercial production environments. The underlying molecular mechanisms associated with bioactive compounds were not explored in detail, and long-term effects of FCP supplementation on productivity and health were not assessed. Moreover, sensory attributes and consumer acceptance of meat were not evaluated.

Future research should focus on elucidating the molecular and metabolic pathways underlying the observed effects of FCP, particularly the role of β-glucans, polyphenols, and flavonoids in regulating immunity and lipid metabolism. Studies under commercial farming conditions are warranted to validate these findings at a larger scale. Additionally, investigations into meat quality attributes, shelf life, and consumer acceptability will further support the practical application of FCP. Exploring synergistic effects with probiotics or enzymes may also enhance its efficacy.

In conclusion, FCP is a promising functional feed ingredient that improves growth performance, carcass traits, gut health, immune response, and fatty acid composition in broilers without adverse effects on physiological organs. Its utilization provides both economic and environmental benefits, supporting sustainable poultry production systems.

## DATA AVAILABILITY

The supplementary data can be made available from the corresponding author upon request.

## AUTHOR’S CONTRIBUTIONS

NN: Implemented the research, statistical analysis, and drafted and revised the manuscript. JB and DR: Statistical analysis and revised the manuscript. MM: Statistical analysis. RKR: Statistical analysis and revised the manuscript. HH, FZA, and MRA: Conducted the research and statistical analysis. All authors have reviewed and approved the final version of the manuscript.

## References

[1] Ogbuewu IP, Mabelebele M, Mbajiorgu CA (2023). Meta-analysis of blood indices and production physiology of broiler chickens on dietary fermented cassava intervention. Trop Anim Health Prod.

[2] Li J, Tao L, Zhang R, Yang G (2021). Effects of fermented feed on growth performance, nutrient metabolism, and cecal microflora of broilers. Anim Biosci.

[3] Nuraini, Nur YS, Djulardi A (2020). Response of laying quail to a diet enriched with cocoa pods fermented by *Pleurotus ostreatus*. J World Poult Res.

[4] Zhu X, Tao L, Liu H, Yang G (2023). Effects of fermented feed on growth performance, immune organ indices, serum biochemical parameters, cecal odorous compound production, and the microbiota community in broilers. Poult Sci.

[5] Ogbuewu IP, Mbajiorgu CA (2024). Enhancement of nutritional and functional qualities of tropical leaf meal as feed ingredients in chickens through the use of fermentation technology. Trop Anim Health Prod.

[6] Xie L, Zhang Y, Liu Z, Li X (2016). Fermentation using the fungus *Pleurotus ostreatus* is crucial for lowering the fiber content. J Food Sci.

[7] Törős G, El-Ramady H, Prokisch J, Llanaj X, Nguyen DHH, Peles F (2023). Modulation of the gut microbiota with prebiotics and antimicrobial agents from *Pleurotus ostreatus* mushroom. Foods.

[8] Setiawan H, Jingga ME, Saragih HT (2018). The effect of cashew leaf extract on small intestine morphology and growth performance of Jawa Super chicken. Vet World.

[9] Prihambodo TR, Sholikin MM, Qomariyah N, Jayanegara A, Batubara I, Utomo DB (2021). Effects of dietary flavonoids on performance, blood constituents, carcass composition and small intestinal morphology of broilers: A meta-analysis. Asian-Australas J Anim Sci.

[10] Tan Z, Halter B, Liu D, Gilbert ER, Cline MA (2022). Dietary flavonoids as modulators of lipid metabolism in poultry. Front Physiol.

[11] Ullah A, Munir S, Badshah SL, Khan N, Ghani L, Poulson BG (2020). Important flavonoids and their role as a therapeutic agent. Molecules.

[12] Abdel-Moneim AE, Shehata AM, Alzahrani SO, El-Sayed ME (2020). The role of polyphenols in poultry nutrition. J Anim Physiol Anim Nutr.

[13] Wang J, Zheng Z, Yang H, Chen J, Xiao Y, Ji X (2022). Effect of β-1,3/1,6-glucan on gut microbiota of yellow-feathered broilers. AMB Express.

[14] Törős G, El-Ramady H, Béni Á, Peles F, Gulyás G, Czeglédi L (2024). *Pleurotus ostreatus* mushroom: A promising feed supplement in poultry farming. Agriculture.

[15] Wijaya A, Putra S (2021). The effect of β-glucan from *Pleurotus ostreatus* on the morphology of the small intestine and the immune system of chickens. J Peternak Indones.

[16] Chen Y, Lee S, Zhang Q (2023). Effects of concentrate feed supplementation on ruminal fermentation and nutrient digestibility in beef cattle. Anim Feed Sci Technol.

[17] Atlı B, Eraslan G, Kılıç S (2019). Solid state fermentation optimization of *Pleurotus ostreatus* for lovastatin production. J Ferment Bioeng.

[18] Ayu PPID, Dewa S (2021). Lactic acid bacteria (*Lactobacillus bulgaricus* and *Streptococcus thermophilus*) in yogurt inhibit the growth of *Escherichia coli*, *Salmonella* Typhimurium, and *Shigella* sp. *in vitro*. J Kesehat Bina Sejahtera.

[19] Yaqoob MU, Wang G, Wang M (2022). An updated review on probiotics as an alternative of antibiotics in poultry—A review. Anim Biosci.

[20] Subagio A (2022). Building the nation with cassava. The prospects of cassava as a high-quality culinary ingredient. Technical guidance and socialization of food crops. Ministry of Agriculture Republic of Indonesia;.

[21] Central Statistics Agency (CSA) of West Sumatra Province (2022). Fruit and vegetable production. Food Crops, Horticulture, and Plantation Service;.

[22] Nurlaeni L (2022). The potential of cassava peels as feed for broiler chickens. J Nutr Ternak Trop Ilmu Pakan.

[23] Wang J, Yao L, Su J, Fan R, Zheng J (2023). Effects of *Lactobacillus plantarum* and its fermentation products on growth performance, immune function, intestinal pH, and cecal microorganisms of Lingnan yellow broilers. Poult Sci.

[24] Animashahun RA, Odebunmi AO, Ojo OA, Adeyemi OT, Adefolalu OO (2024). Utilization of solid state fermented cassava peel-leaf mix meal as a substitute for maize in broiler chickens' diets. Afr J Res Anim Vet Sci.

[25] Leeson S, Summers JD (2005). Commercial poultry nutrition.

[26] Hafid H (2022). Growth and development of chicken carcass in different sexes and ages. Indones J Agric Res.

[27] Hwang J, Lee JH, Kim YJ, Hwang I, Kim YY, Kim HS (2024). Highly accurate measurement of the relative abundance of oral pathogenic bacteria using colony-forming unit-based qPCR. J Periodontal Implant Sci.

[28] AOAC (2016). Official methods of analysis. Virginia: Association of Official Analytical Chemists;.

[29] Sharmin F, Sarker MSK, Chun J (2016). Cholesterol contents in raw and cooked chicken meat through a validated method. Asian-Australas J Biosci Biotechnol.

[30] Azzahra YR, Toharmat T, Prihantoro I (2022). Bio-processing plantation by-products with white oyster mushroom (*Pleurotus ostreatus*) to improve fermentability and digestibility. Bul Peternak.

[31] Olagunju LK, Isikhuemhen OS, Dele PA, Anike FN, Essick BG, Holt NW (2023). *Pleurotus ostreatus* can significantly improve the nutritive value of lignocellulosic crop residues. Agriculture.

[32] Yanuartono Y, Purnamaningsih H, Indarjulianto S, Nururrozi A (2017). The potential of straw as feed for ruminants. J Ilmu Ilmu Peternak.

[33] Irwan A, Riyanti (2019). Bioconversion of palm oil mill effluent into larval biomass of black soldier fly (*Hermetia illucens*) as alternative protein source. J Insects Food Feed.

[34] Teufack S, Noumbissi MNB, Ngouana TR, Tchouan Deffo G, Edie NLW, Taboumda E (2024). Effects of fermented palm kernel cake in acidic and basic solutions on the performance of broiler chickens. Online J Anim Feed Res.

[35] Ibarra-Rondón A, Durán-Sequeda D, Castro-Pacheco AC, Castilla PJF, Rosales RB, Mojica-Rodríguez JE (2025). Optimization of palm kernel cake bioconversion with *P ostreatus*: An efficient lignocellulosic biomass value-adding process for ruminant feed. Fermentation.

[36] Asriyanti IN, Hutabarat J, Herawati VE (2018). The effect of fermented *Lemna* sp flour in artificial feed on feed utilization, growth, and survival rate of dumbo catfish fry (*Clarias gariepinus*). E-J Rekayasa Teknol Budidaya Perairan.

[37] Gopi M, Dutta N, Pattanaik AK, Jadhav SE, Madhupriya V, Tyagi PK (2020). Effect of polyphenol extract on performance, serum biochemistry, skin pigmentation and carcass characteristics in broiler chickens fed with different cereal sources under hot-humid conditions. Saudi J Biol Sci.

[38] Nguyen TT, Nguyen DT, Nguyen VC (2021). Effects of fermented feed with *Pleurotus ostreatus* on growth performance and nutrient digestibility in broiler chickens. J Appl Poult Res.

[39] Khan SH, Khan MA, Khan RU (2023). Fermented wheat bran with *Pleurotus ostreatus* improves growth performance and gut health in broiler chickens. Animals.

[40] Alagbe JO, Oke OL (2022). Fermentation of rice bran with *Pleurotus ostreatus* enhances broiler growth performance and nutrient utilization. Trop Anim Health Prod.

[41] Raji MO, Oloko AB, Fasasi MO, Bamgbose AM, Oso AO (2021). Internal organs weight of broiler chicken fed graded levels of sundried cassava peels meal as a replacement of wheat bran. Niger J Anim Prod.

[42] Kwon HK, Jo W, Park HJ (2018). Immune-enhancing activity of *C militaris* fermented with *Pediococcus pentosaceus* in CY-induced immunosuppressed model. BMC Complement Altern Med.

[43] Anwar MI, Tahir M, Awais MM, Raza A, Akhtar M, Muhammad F (2023). Purification, characterization and protective effects of indigenous yeast derived β-glucans against salmonellosis in broilers. Pak Vet J.

[44] Sari YW, Budiarti A, Ramadhan SD (2020). Effects of fermented oyster mushroom (*Pleurotus ostreatus*) on immune organs in broiler chickens. Int J Poult Sci.

[45] Apperson KD, Cherian G (2017). Effect of whole flax seed and carbohydrase enzymes on gastrointestinal morphology, muscle fatty acids, and production performance in broiler chickens. Poult Sci.

[46] Šimić A, González-Ortiz G, Mansbridge SC, Rose SP, Bedford MR, Yovchev D (2023). Broiler chicken response to xylanase and fermentable xylooligosaccharide supplementation. Poult Sci.

[47] Czech A, Grela ER, Kiesz M (2021). The effect of fermented feed components on the intestinal morphology and microbiota of poultry—A review. Animals.

[48] Pinzón-Osorio CA, Álvarez-Mira DM, Betancourt L (2023). Effect of the inclusion of *Ganoderma* spp on gut morphometry and growth performance of broiler chickens. Rev Bras Zootec.

[49] Inatomi T, Otomaru K (2018). Effect of dietary probiotics on the semen traits and antioxidative activity of male broiler breeders. Sci Rep.

[50] Sutthisa W, Anujakkawan S (2023). Antibacterial potential of oyster mushroom (*Pleurotus ostreatus*) extract against pathogenic bacteria. J Pure Appl Microbiol.

[51] Yadav S, Jha R (2019). Strategies to modulate the intestinal microbiota and improve gut health of broiler chickens through plant bioactives. Anim Feed Sci Technol.

[52] Manafi M, Khalaji S, Hedayati M, Pirany N (2017). Efficacy of *Bacillus subtilis* and bacitracin methylene disalicylate on growth performance, digestibility, blood metabolites, immunity, and intestinal microbiota after intramuscular inoculation with *Escherichia coli* in broilers. Poult Sci.

[53] Bormon C, Akib G, Rifat A, Hossain M, Uddin N, Hossain F (2024). Effects of oyster mushroom (*Pleurotus ostreatus*) stem residue supplementation on growth performance, meat quality and health status of broilers. Poult Sci.

[54] Nasir J, Chand N, Naz S, Alhidary I, Khan R, Batool S (2023). Dietary oyster mushroom (*Pleurotus ostreatus*) waste inhibits experimentally induced *Eimeria tenella* challenge in Japanese quails model. Animals.

[55] Effiong M, Umeokwochi C, Afolabi I, Chinedu S (2024). Comparative antioxidant activity and phytochemical content of five extracts of *Pleurotus ostreatus*. Sci Rep.

[56] Hamad D, El-Sayed H, Ahmed W, Sonbol H, Ramadan M (2022). GC-MS analysis of potentially volatile compounds of *Pleurotus ostreatus* polar extract: *In vitro* antimicrobial, cytotoxic, immunomodulatory, and antioxidant activities. Front Microbiol.

[57] Ghaffari A (2015). The effect of statin and mushroom beta-glucan on lipid profile in broiler chickens. J Anim Physiol Anim Nutr.

[58] Sogunle OM, Labinjo OS, Olanite JA, Adebowani AA (2019). Rendimiento del crecimiento y perfil de la sangre de gallos jóvenes por la administración del champiñón ostra (*Pleurotus ostreatus*) en agua y alimento. Arch Zootec.

[59] Zhang T, Yuan D, Xie J, Lei Y, Li J, Fang G (2019). Evolution of the cholesterol biosynthesis pathway in animals. Mol Biol Evol.

[60] Gomes RS, Martins TP, Pereira LM, Costa ML, Ferreira AB (2022). β-glucans and polyphenol enrichment in chicken thigh meat through *Pleurotus ostreatus* supplementation. Nutrients.

[61] Pereira LM, Gomes RS, Costa ML, Ferreira AB, Martins TP (2024). Enhancing fatty acid profiles in poultry with *Pleurotus ostreatus* fermentation. Food Chem.

[62] Rodrigues FA, Pereira LM, Silva JK, Sousa EC, Almeida RF (2023). Omega fatty acids in fermented mushroom-supplemented chicken meat. Nutrients.

[63] Prates JAM (2025). The role of meat lipids in nutrition and health: Balancing benefits and risks. Nutrients.

